# Scaffold attachment factor B2 (*SAFB2*)-null mice reveal non-redundant functions of *SAFB2* compared with its paralog, *SAFB1*

**DOI:** 10.1242/dmm.019885

**Published:** 2015-09-01

**Authors:** Shiming Jiang, Tiffany A. Katz, Jason P. Garee, Francesco J. DeMayo, Adrian V. Lee, Steffi Oesterreich

**Affiliations:** 1 Department of Molecular and Cellular Biology, Baylor College of Medicine, One Baylor Plaza, Houston, TX 77030, USA; 2 Lester and Sue Smith Breast Center, Baylor College of Medicine, One Baylor Plaza, Houston, TX 77030, USA

**Keywords:** Reproduction, Scaffold attachment factor B2 (SAFB2), Sertoli cells

## Abstract

Scaffold attachment factors SAFB1 and SAFB2 are multifunctional proteins that share >70% sequence similarity. *SAFB1*-knockout (*SAFB1^−/−^*) mice display a high degree of lethality, severe growth retardation, and infertility in male mice. To assess the *in vivo* role of SAFB2, and to identify unique functions of the two paralogs, we generated *SAFB2*^−/−^ mice. In stark contrast to *SAFB1*^−/−^, *SAFB2*^−/−^ offspring were born at expected Mendelian ratios and did not show any obvious defects in growth or fertility. Generation of paralog-specific antibodies allowed extensive expression analysis of SAFB1 and SAFB2 in mouse tissues, showing high expression of both SAFB1 and SAFB2 in the immune system, and in hormonally controlled tissues, with especially high expression of SAFB2 in the male reproductive tract. Further analysis showed a significantly increased testis weight in *SAFB2*^−/−^ mice, which was associated with an increased number of Sertoli cells. Our data suggest that this is at least in part caused by alterations in androgen-receptor function and expression upon deletion of *SAFB2*. Thus, despite a high degree of sequence similarity, *SAFB1*^−/−^ and *SAFB2*^−/−^ mice do not totally phenocopy each other. *SAFB2*^−/−^ mice are viable, and do not show any major defects, and our data suggest a role for SAFB2 in the differentiation and activity of Sertoli cells that deserves further study.

## Abstract (precis)

**Summary:** Analysis of *SAFB2^−/−^* mice reveals lack of redundancy between the closely related paralogs *SAFB1* and *SAFB2*, and a role for SAFB2 in the male reproductive system, specifically in Sertoli cells.

## INTRODUCTION

Scaffold attachment factor B2 (SAFB2) is a member of a protein family that also contains the paralog SAFB1 and a closely related protein scaffold attachment factor-like transcriptional modulator (SLTM). SAFB2 was originally identified as KIAA0138 and subsequently named SAFB2 based on its high homology to SAFB1 (Oesterreich, 2003; Townson et al., 2003; Jiang et al., 2006; Hammerich-Hille et al., 2009, 2010; Hong et al., 2012). The two proteins display the highest conservation in the functional SAF-Box and RNA recognition motif (RRM) domains. In the human genome, *SAFB1* and *SAFB2* map adjacent to each other to chromosome 19p13.3, where they are oriented in a bidirectional divergent configuration and separated by an ∼500 bp region that can act as a bidirectional promoter (Townson et al., 2003). Given the high degree of sequence similarity, we hypothesized that the proteins have both shared but also unique functions.

There have been many reports on SAFB1, but less is known about SAFB2. SAFB1 is a multifunctional protein that binds both DNA and RNA and is involved in the attachment of chromatin to the nuclear matrix, and in the regulation of transcription, the stress response and splicing (Garee and Oesterreich, 2010; Garee et al., 2011; Altmeyer et al., 2013). More recently it has been shown that SAFB1 might play a role in embryonic stem (ES)-cell self-renewal as a target of FoxD3 (Plank et al., 2014). Intriguingly, there have been a number of reports that collectively imply an important role of SAFB1 in DNA damage pathways. Lachepelle et al. demonstrated that SAFB1 binds directly to Werner syndrome helicase (Wrn), which is important for DNA repair and replication (Lachapelle et al., 2011). These studies also showed that Wrn protein was required for immortalization and tumorigenesis in *SAFB1*^−/−^ models. SAFB1 also interacts with, and downregulates, p53, resulting in suppression of p53-mediated gene expression (Peidis et al., 2011). Further demonstrating a role in DNA damage signaling, γH2AX spreading is enabled by SAFB1 binding to acetylated histones (Altmeyer et al., 2013). Reactive oxygen species (ROS) generation is indirectly regulated by SAFB1, with knockdown inducing the expression of xanthine oxoreductase, a key enzyme in ROS production (Lin et al., 2008).

Both SAFB1 and SAFB2 have been shown to interact with and repress transcriptional activity of nuclear receptors, including estrogen receptor alpha (ERα) (Townson et al., 2003; Hashimoto et al., 2012). Similar to SAFB1, SAFB2 can block cell growth, because SAFB2 overexpression leads to a decrease in the proportion of cells in S phase and an increase in cells blocked in G_1_ (Townson et al., 2003). SAFB2 also plays a role in alternative RNA splicing, inhibiting the splicing of a tra2β variable exon (Sergeant et al., 2007), and binding to and regulating activity of the SR protein kinases SRPK1 and SRPK1a (Tsianou et al., 2009). More recently, it was shown that SAFB2 is a substrate for the E3 ubiquitin ligase activity of BRCA1 (also known as BARD), resulting in regulation of SAFB2 protein expression (Song et al., 2011).

To identify unique physiological functions of SAFB2, we generated *SAFB2*^−/−^ mice through targeted deletion in ES cells. Interestingly, *SAFB2*^−/−^ offspring were born at expected Mendelian ratios and did not show any obvious defects in growth or fertility. Despite many shared functions, the presence of severe phenotypes of *SAFB1*^−/−^ mice, including a high degree of lethality, severe growth retardation, and infertility in male mice, indicate that SAFB2 cannot fully compensate for loss of SAFB1 (Ivanova et al., 2005). Extensive expression analysis revealed high SAFB2 expression in the male reproductive system, and subsequent focused analysis showed that *SAFB2*^−/−^ mice have significantly bigger testes, and a significant increase in Sertoli cell number, and we show data suggesting that this is at least in part a result of altered androgen receptor (AR) activity. Our results reveal physiologically diverse functions of SAFB1 and SAFB2, including a unique role of SAFB2 in Sertoli cells.

**TRANSLATIONAL IMPACT**

**Clinical issue**

Scaffold attachment binding factors (SAFBs) are a family of multifunctional proteins that include SAFB2, SAFB1 and the scaffold attachment factor-like transcriptional modulator (SLTM). SAFBs have been shown to play a role in RNA splicing, DNA damage signaling and transcription, and are also involved in some forms of cancer. In addition, *SAFB1^–/–^* mice display severe growth retardation, as well as deficits in reproductive function. Male *SAFB1^–/–^* mice are sterile and a significant amount of lethality is observed in both prenatal and neonatal pups. Whereas many mechanisms of reproductive dysfunction exist, reproductive incompetence in humans still remains unexplained. The SAFB family might play an important role in reproductive function and infertility.

**Results**

In this study, the authors generated *SAFB2^–/–^* mice to determine whether the two paralogs, SAFB1 and SAFB2, have redundant or distinct functions. They find that indeed the two proteins have likely distinct functions. *SAFB2^–/–^* mice do not display the severe growth retardation or significant neonatal lethality of the *SAFB1^–/–^* animals. SAFB2 is likely to play a role in male reproduction because it was found to be highly expressed throughout the male reproductive track and to be involved in the regulation of the androgen receptor (AR). Additionally, *SAFB2^–/–^* mice showed a significantly increased testis weight and a higher number of Sertoli cells in the testes, compared with wild type. Finally, the study includes a comprehensive expression analysis of SAFB1 and SAFB2 in mouse tissues, showing that they have shared but also unique target tissues.

**Implications and future directions**

SAFB1 and SAFB2 play important roles in a number of normal and pathophysiological processes. This study shows that loss of SAFB2 has fewer deleterious effects compared to loss of SAFB1, but analysis of the *SAFB2^–/–^* phenotypes suggests a role for SAFB2 in the male reproductive system. This *SAFB2^–/–^* mouse model provides a unique system to study SAFB2 function in the normal male reproductive system, as well as in pathophysiological conditions such as cancer.

## RESULTS

### Generation of *SAFB2***^−/−^** mice

**Fig. 1.**
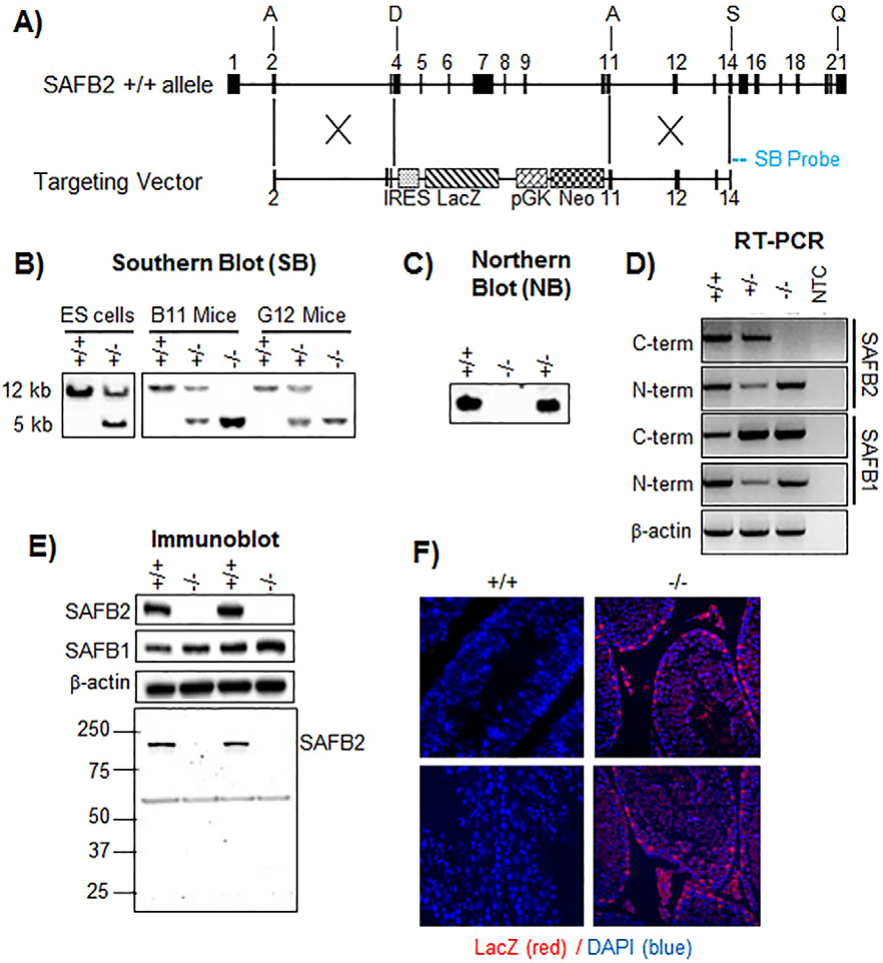
**Generation of *SAFB2*^−/−^ mice.** (A) *SAFB2* mouse allele, and targeting construct for deletion of the *SAFB2* genomic fragment from exons 4 to 10. SB, Southern blot probe. (B) Southern blot analysis of genomic DNA from embryonic stem (ES) cells (left panel) and mice (right panel). *SAFB2*^+/+^ and *SAFB2*^−/−^ bands have sizes of 12 kb and 5 kb, respectively. (C) Northern blot analysis of RNA from *SAFB2*^+/+^, *SAFB2*^+/−^ and *SAFB2*^−/−^ mice. (D) RT-PCR using RNA from *SAFB2*^+/+^, *SAFB2*^+/−^ and *SAFB2*^−/−^ mice and primers targeting both the N and C terminal regions of SAFB2 and SAFB1. NTC, non-template control. (E) Immunoblot of lysates from either *SAFB2*^+/+^ or *SAFB2*^−/−^ mice with antibodies targeting SAFB2 and SAFB1. Lower panel shows an entire SAFB2 immunoblot to provide proof of expression of the N-terminal fragment. (F) LacZ (β-galactosidase) staining of *SAFB2*^+/+^ or *SAFB2*^−/−^ Sertoli cells. Blue, DAPI; red, Alexa Fluor 546 (*lacZ*).

*SAFB2*^−/−^ mice were generated by homologous recombination in 129/Sv ES cells, replacing exons 4-10 with an IRES-lacZ-SV40pA (β-galactosidase) marker and a PGK-Neomycin-bGHpA selection marker (Fig. 1A). The targeting construct was linearized and electroporated into ES cells, and the neomycin-resistant ES cell colonies were screened by Southern blot analysis (Fig. 1B, left panel). Three recombinant clones were injected into C57BL/6 blastocysts, and two (B11 and G12) were transmitted to germ line. The chimeric mice were bred with wild-type C57BL/6 mice to produce F1 mixed background (129/Sv and C57BL/6) heterozygous mutant mice (*SAFB2*^+/−^). Heterozygous F1 mice from each line were intercrossed to produce two independent lines of homozygous mutant mice (*SAFB2*^−/−^). Genotyping was confirmed by Southern blot analysis (Fig. 1B, right panel). Loss of *SAFB2* mRNA expression was confirmed by northern blot analysis (Fig. 1C), and by RT-PCR assays using primers spanning the *Safb2* C-terminus (exons 18-21) (Fig. 1D). RT-PCR with primers covering exons 4-7 revealed remaining expression of the N-terminus. *SAFB1* mRNA expression was not affected, as shown by RT-PCR using primers spanning N-terminal (exons 1-4) and C-terminal (exons 9-11) regions of the gene (Fig. 1D).

To determine whether the remaining expression of N-terminal RNA product would result in expression of a truncated protein, we generated polyclonal antibodies against the SAFB2 N-terminus (aa105-199, exons 3-5) (Materials and Methods, and supplementary material Fig. S1). We were unable to detect full-length or truncated SAFB2 in the *SAFB2*^−/−^ mice, even after prolonged exposure of the film, suggesting that either there is no truncated N-terminal protein expression or the potential truncated protein is highly unstable (Fig. 1E). As expected, SAFB1 protein expression was detected in both SAFB2^+/+^ and *SAFB2*^−/−^ mice, confirming specificity of the *SAFB2* knockout. Finally, immunofluorescence for β-galactosidase was performed on testes from *SAFB2*^+/+^ and *SAFB2*^−/−^ mice, indicating the presence of β-galactosidase in *SAFB2*^−/−^ Sertoli cells, and also in the germ cells although at a lower level (Fig. 1F). We conclude that our model represents a *SAFB2*-null mutation resulting in lack of SAFB2 protein expression.

### *SAFB2*^−/−^ mice do not display any gross phenotypes

**Fig. 2.**
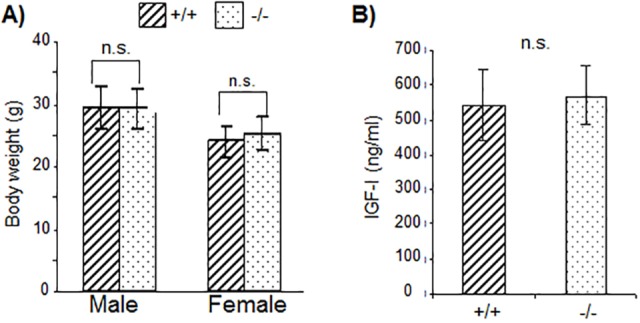
**Body weight and IGF-I levels in *SAFB2*^−/−^ mice.** (A) Body weight measurements in male (*n*=5) and female (*n*=6) *SAFB2*^+/+^ and *SAFB2*^−/−^ mice, presented as mean±s.e.m. (B) Serum IGF-I levels (mean±s.e.m.) in *SAFB2*^+/+^ (*n*=10) and *SAFB2*^−/−^ (*n*=10) mice.

We have previously shown that *SAFB1*^−/−^ mice display both prenatal and neonatal lethality, growth retardation (caused by defects in the IGF signaling system), infertility in male mice and reduced testis weight (Ivanova et al., 2005). To determine whether *SAFB2*^−/−^ mice displayed similar defects, we first assessed if there was some degree of lethality by analyzing genotypes of litters from *SAFB2*^+/−^ intercrosses. All three genotypes of *SAFB2* (^+/+^, ^+/−^ and ^−/−^) were produced at the expected Mendelian distribution (1:2:1 ratio), suggesting that *SAFB2*^−/−^ did not affect viability (Table 1). There were also no significant differences in body weight (Fig. 2A) or levels of circulating IGF-1 (Fig. 2B) between *SAFB2*^+/+^ and *SAFB2*^−/−^ mice.

**Table 1.**
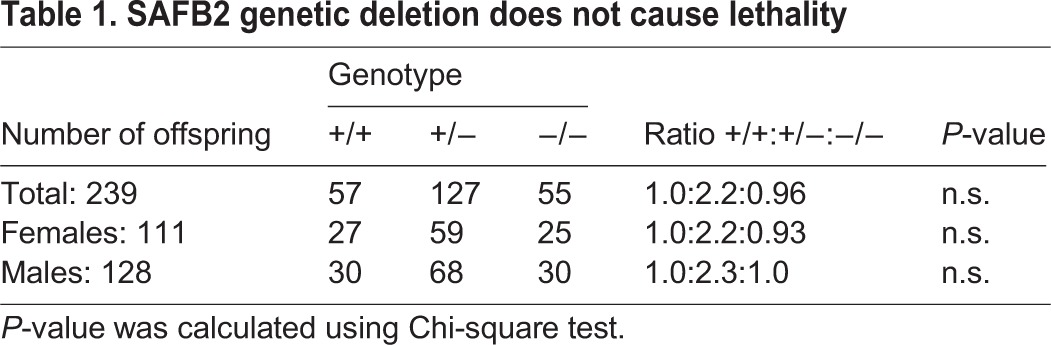
**SAFB2 genetic deletion does not cause lethality**

To test whether *SAFB2*^−/−^ caused defects in fertility, we bred *SAFB2*^−/−^ males with *SAFB2*^+/+^ females, and *SAFB2*^−/−^ females with *SAFB2*^+/+^ males. The number of successful pregnancies, number of litters and number of pups per litter were not significantly different (Table 2), suggesting that there are no major defects in the development of the reproductive system. Gross histological analysis of *SAFB2*^−/−^ mice also did not identify any major structural defects in brain, spine, skin, tonsil, lung, heart, liver, spleen, stomach, intestine and kidney (data not shown). Thus, in stark contrast to *SAFB1*^−/−^ mice, *SAFB2*^−/−^ does not cause any major phenotypes.

**Table 2.**
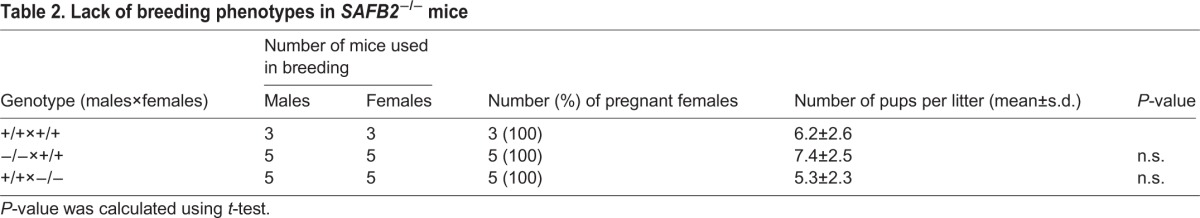
**Lack of breeding phenotypes in *SAFB2*^−/−^ mice**

### SAFB2 protein is highly expressed in the male reproductive tract, and *SAFB2*^−/−^ results in increased testis weight and number of Sertoli cells

**Fig. 3.**
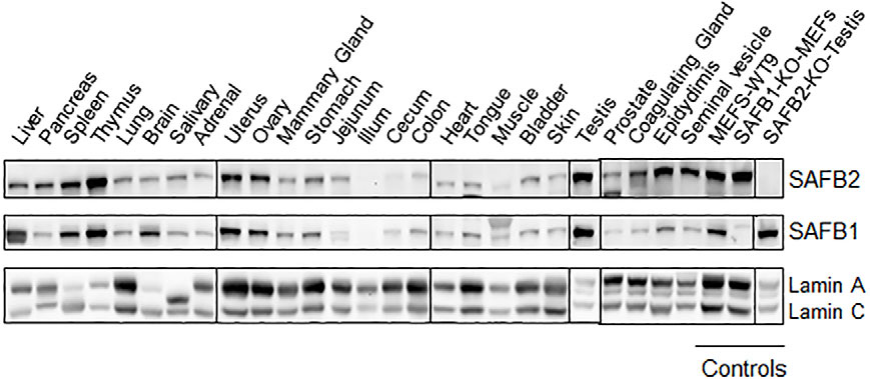
**SAFB2 and SAFB1 protein expression levels in mouse tissues.** Immunoblot analysis using antibodies against SAFB2, SAFB1 and Lamin A/C (as loading control). KO, knock out.

**Fig. 4.**
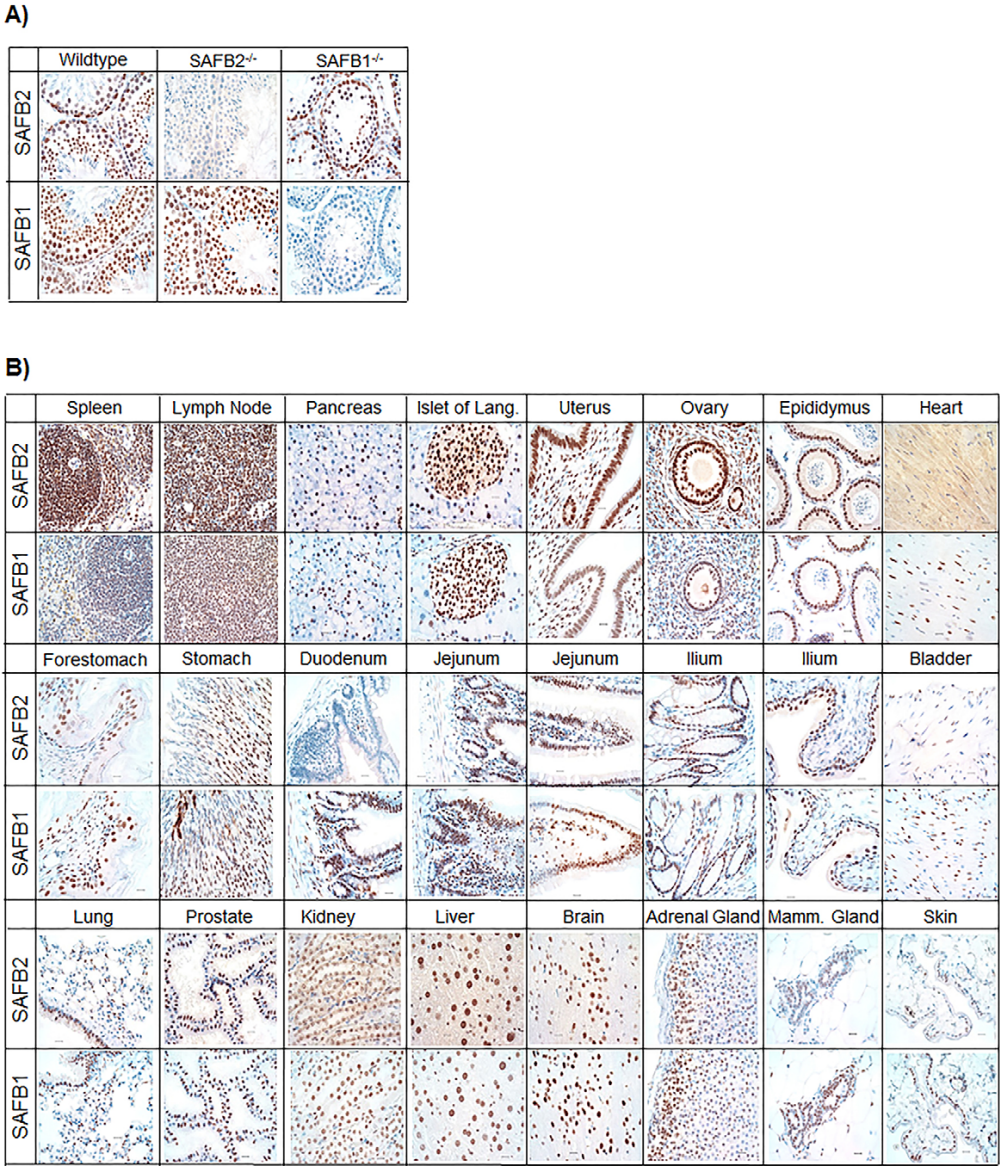
**Immunohistochemical staining of SAFB2 and SAFB1 in mouse tissues.** (A) SAFB2 and SAFB1 IHC using mouse testes from wild-type, *SAFB2*^−/−^ and *SAFB1*^−/−^ mice. Tissues were counterstained with hematoxylin. (B) IHC for SAFB2 and SAFB1 in mouse tissues, as indicated (scale bars: 20 μm).

To narrow down which tissue could have potential, less obvious, phenotypes in the *SAFB2*^−/−^ mice, we surveyed a large panel of mouse tissues for SAFB2 protein, and compared it to that of SAFB1. Immunoblot (Fig. 3) and immunohistochemistry (IHC) (Fig. 4) analyses were performed, using tissue from knockout animals as controls (Fig. 4A). Immunoblot data showed high expression of both SAFB1 and SAFB2 in the immune system (spleen, thymus) and in hormonally regulated organs (uterus, ovary). Of interest, in some tissues of the male reproductive tract, including the prostate, coagulating gland, epididymus and seminal vesicle, SAFB2 expression was significantly higher than that of SAFB1. In general, protein expression was concordant between immunoblot and IHC analyses, and IHC data revealed a potential weak cytoplasmic location of SAFB2 in some tissues, such as heart and kidney. These results indicate that SAFB2 might play a role in the male reproductive system.

**Fig. 5.**
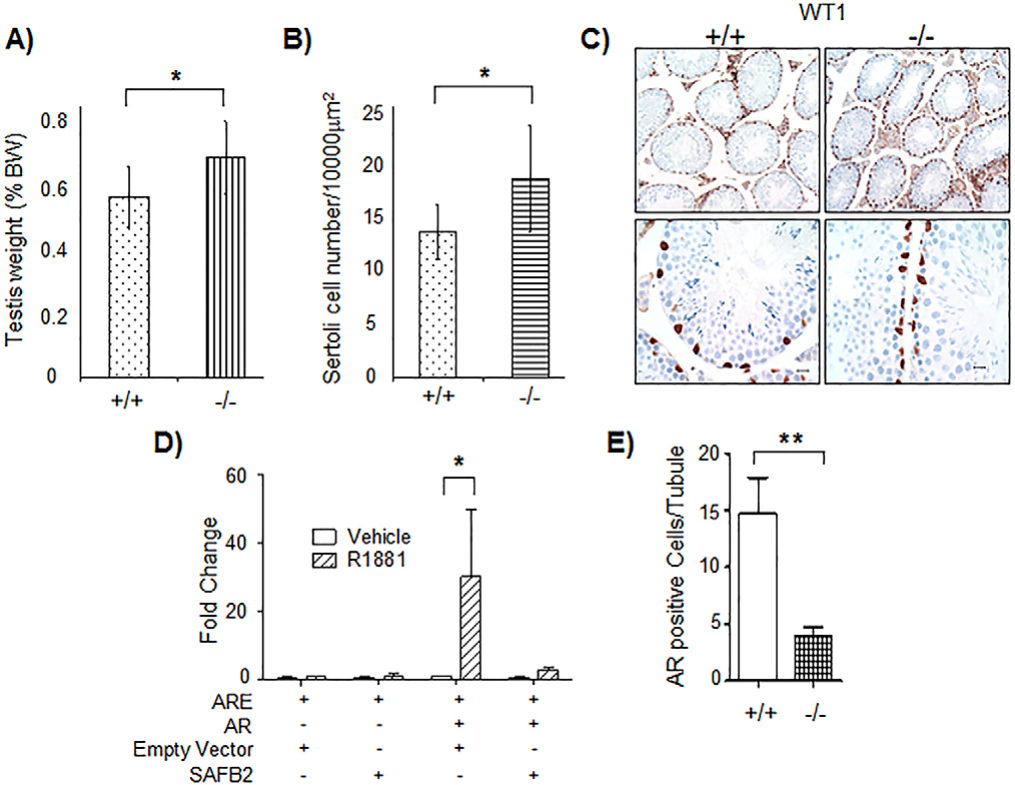
**Increased number of Sertoli cells in *SAFB2*^−/−^ mice.** (A) Bars represent testis weight as percent (mean±s.d.) of total body weight (BW), measured in *SAFB2*^+/+^ (*n*=12) and *SAFB2*^−/−^ (*n*=22) mice. (B) Number of Sertoli cells (mean±s.d.) in *SAFB2*^+/+^ (*n*=4) and *SAFB2*^−/−^ (*n*=14) mice. (C) Representative picture of IHC for Wilms tumor 1 (WT1) in *SAFB2*^+/+^ and *SAFB2*^−/−^ mouse testes (scale bars: 10 μm). (D) Reporter assay in LNCaP cells (mean±s.e.m., *n*=4) with fold change compared to ARE alone. A two-way ANOVA with a Bonferroni post-test was used to analyze the data (*P<0.05). (E) Number of AR-positive cells/tubule (mean±s.e.m., *n*=6). A *t*-test was conducted for statistical analysis (**P<0.01).

Despite lack of gross phenotypes on fertility, further analysis showed that *SAFB2*^−/−^ mice displayed a significant increase in testis weight compared to *SAFB2*^+/+^ mice (Fig. 5A). This was in contrast to *SAFB1*^−/−^ male mice, which we had previously shown to have decreased testes size (Ivanova et al., 2005). A potential cause for increased testes size in *SAFB2*^−/−^ mice were changes in Sertoli cells, known as the ‘nursing’ cells of the testes. Staining for expression of Wilms tumor 1 (WT1), a marker of Sertoli cells, showed a significantly increased number of Sertoli cells in *SAFB2*^−/−^ testes compared to *SAFB2*^+/+^ testes (Fig. 5B). A representative section showing increased WT1 staining in the *SAFB2*^−/−^ mice is shown in Fig. 5C. Because SAFB1 was previously shown to regulate AR activity (Mukhopadhyay et al., 2014) and AR signaling can effect numbers of Sertoli cells (Johnston et al., 2004; Tan et al., 2005), we next tested the effect of SAFB2 on androgen-stimulated AR activity in a number of cell lines (LNCaP, COS7, 239T). We observed significant repression of AR activity by SAFB2 overexpression in LNCaP (Fig. 5D) and COS7 cells, but not in 293T (data not shown). In adult mice (12 months), there was less AR expression in *SAFB2*^−/−^ testes compared to *SAFB2*^+/+^ testes (Fig. 5E, and supplementary material Fig. S2), possibly as a result of negative feedback as previously described (Cai et al., 2011). There were no differences in expression of ERβ between *SAFB2*^−/−^ and *SAFB2*^+/+^ testes (data not shown). Thus, *SAFB2*^−/−^ mice have increased weight of testes, associated with an increase in Sertoli cell number, likely at least in part owing to altered AR signaling.

## DISCUSSION

SAFB2 was originally identified based on high homology with its paralog, SAFB1 (Townson et al., 2003). Similar to SAFB1, SAFB2 is a multifunctional protein with roles in transcriptional repression, RNA splicing and SR protein kinase signaling, and with effects on cell growth (Tsianou et al., 2009; Garee and Oesterreich, 2010; Garee et al., 2011; Song et al., 2011; Altmeyer et al., 2013). Here, we describe a novel mouse model deficient in SAFB2. In contrast to *SAFB1*^−/−^ mice, which have major phenotypes, including prenatal and neonatal lethality, growth retardation and fertility problems, there were no gross phenotypes in *SAFB2*^−/−^ mice. Comprehensive expression analysis of both paralogs shows high expression of SAFB2 in the male reproductive tract, and our results indicate a role for SAFB2 in the testes.

*SAFB1*^−/−^ mice display severe defects, including a high rate of lethality and dwarfism, and, additionally, the males are infertile (Ivanova et al., 2005). These dramatic phenotypes were not recapitulated with genetic deletion of *SAFB2*, suggesting that either SAFB1 has unique critical functions not shared with SAFB2, or that SAFB1 can fully compensate for loss of SAFB2, whereas the reverse is not true. This might indicate that, although SAFB1 and SAFB2 are paralogs, having arisen from a common ancestor, they might not have retained all their shared functions, and new functions have been gained. Previous work has demonstrated that SAFB1 and SAFB2 can heterodimerize, but only SAFB1 is able to homodimerize (Townson et al., 2003). Elegant biochemical work by Sergeant et al. (2007) has clearly shown that SAFB1 is a component of a small nuclear complex, whereas SAFB2 is part of higher molecular mass nuclear protein complexes. There are also differences in subnuclear localization between SAFB1 and SAFB2 that are cell-type-dependent, and additional studies are necessary to gain a more detailed understanding of the shared and unique functions of the paralogs.

In our study, we provide the first comprehensive expression analysis of SAFB1 and SAFB2, showing high expression of both proteins in the immune system and in hormonally regulated organs. *SAFB1*^−/−^ mice have defects in the immune system (S.O. et al., our unpublished data) but, again, this phenotype is not mirrored in *SAFB2*^−/−^ mice. Expression of SAFB2 was especially high in the male reproductive tract, in agreement with previous studies showing high SAFB2 expression in Sertoli cells (Sergeant et al., 2007). SAFB1 is also highly expressed in the testes, and has been shown to be involved in the regulation of AR function, indicating that SAFB1 might also play a role in the testes (Mukhopadhyay et al., 2014). Upon detailed analysis, we found an increased weight of the testes in *SAFB2*^−/−^ male mice. We hypothesize that this is at least in part the result of SAFB2 playing a role in Sertoli cells, because genetic deletion of SAFB2 led to an increase in the number of Sertoli cells.

Androgen signaling controls the regulation of Sertoli cell number and activity (Johnston et al., 2004; Atanassova et al., 2005; Tan et al., 2005; Walker and Cheng, 2005), and, because we show here that SAFB2 can regulate AR activity and levels, it is feasible that this cofactor role of SAFB2 mediates the effect on Sertoli cells. Our recent preliminary studies have shown that AR expression was increased and proliferation was decreased in pre-pubertal testes from *SAFB2*^−/−^ compared to *SAFB1*^+/+^ mice (data not shown), suggesting early maturation in *SAFB2*^−/−^ mice, and this will be the focus of future studies. We cannot exclude the involvement of other pathways, such as altered follicle-stimulating hormone (FSH) and estrogen signaling, because both pathways have been implicated in Sertoli cell functions (Smith et al., 1982; Johnston et al., 2004; Atanassova et al., 2005; Tan et al., 2005; Walker and Cheng, 2005; Lucas et al., 2011). Of note, we did not detect altered ERβ levels in testes when comparing the two genotypes. Finally, SAFB2 might also have specific splicing functions in Sertoli cells, as previously suggested (Sergeant et al., 2007), that contribute to the observed phenotypes. More detailed mechanism-oriented questions are the topic of future studies.

In summary, we have described the mouse phenotype with genetic loss of the multifunctional transcriptional repressor SAFB2. Genetic deletion of SAFB2 shows a mild phenotype with no gross structural defects, growth retardation or reproductive defects. Our data suggest a role for SAFB2 in the male reproductive tract, especially in Sertoli cells, which deserves further investigation. The results of this study provide the basis for future studies to delineate the unique and overlapping physiological function of the two SAFB paralogs, which have important roles in normal and pathobiology.

## MATERIALS AND METHODS

### Gene targeting and generation of *SAFB2*^−/−^ mice

The targeting vector resulting in deletion of exons 4 (amino acid 236) through 11 was generated by amplifying a 4.3-kb region (5′ arm), or a 3.4 kb region (3′ arm), from 129Sv mouse brain tissue genomic DNA using primer set 1 or primer set 2 (supplementary material Table S1). PCR products were then cloned into pGEM 5Zf(+) using *Sac*II/*Not*I sites (3′ arm) or *Xho*I site (5′ arm). An internal ribosome entry site (IRES)-*lacZ* (β-galactosidase)-*neo*-selectable cassette was inserted between the arms. After electroporation of the linearized targeting construct into 129/Sv ES cells, Southern blot analysis was used to screen for homologous recombination in 192 G418-resistant ES clones. A 600-bp probe was cloned from 129Sv genomic DNA and hybridized to *Bam*HI-digested genomic DNA (Fig. 1A, and supplementary material Table S1). Three independent *SAFB2*^−/−^ ES clones were injected into blastocytes (C57BL/6J) and implanted into pseudo-pregnant females. Three lines of *SAFB2*^−/−^ mice were generated, which were analyzed in a C57BL/6J×129/Sv mixed genetic background. The mice used in this study were maintained on a 12-h daylight cycle and fed a diet of a standard food and water *ad libitum* in a pathogen-free facility at Baylor College of Medicine (BCM). Animal care was performed in accordance with BCM institutional guidelines.

### RNA analysis

Total RNA was extracted using the RNeasy Mini Kit (Qiagen), reverse transcribed, and PCR was performed as previously described (Ivanova et al., 2005). *SAFB2* cDNA spanning exons 4 through 7, and 18 through 21, was amplified with primers shown in supplemental material Table S1. To determine specificity of the *SAFB2* knockout, *SAFB1* cDNA spanning exons 1 through 4, and 9 through 11, was amplified. β-actin was used as loading control. For northern blot analysis, total RNA was separated by gel electrophoresis, transferred to a membrane, and then hybridized with a [^32^P]-dCTP-labeled cDNA probe (619 bp) targeting the mouse *SAFB2* C-terminal sequence (position 2666 bp to 3284 bp) (Fig. 1A, and supplementary material Table S1).

### Immunoblot analysis

To analyze SAFB2 expression in mouse tissues and confirm loss of expression of SAFB2 in the *SAFB2*^−/−^ mice, we generated an antibody targeting the mouse SAFB2 N-terminus (amino acids 105 to 199). To generate mouse SAFB2 N-terminal antibody, a cDNA fragment targeting amino acids 105 to 199 (RYGQDGVVILQSSQDRDTMDTGVPDGMEAEDLSVPCLGKADTVNQILHAFDDSKEYVAAQLGQLPAQLLKHAVDEEVFKNTLEASVSDLKVTLAD) was amplified using primer pairs with *Bam*HI and *Eco*RI restriction sites (see supplementary material Table S1 for sequence of primer pairs), and cloned in frame with GST into *Bam*HI/*Eco*RI sites in pGEX-2TK vector. The GST-mB2 N-terminal peptide was expressed in B21 *E. coli* (supplementary material Fig. S1A) and purified with Glutathione Sepharose 4 Fast Flow beads (supplementary material Fig. S1B). Polyclonal serum was generated (Sigma), affinity-purified using the GST-mB2 N-terminal peptide, and verified with immunoblotting (supplementary material Fig. S1C,D).

For the analysis of SAFB1 protein expression, our previously described (Ivanova et al., 2005) polyclonal antibody (B1-1753) was used. Antibodies against β-actin (Sigma A5441, mouse monoclonal) or lamin A/C antibodies (Cell Signaling 2032, rabbit polyclonal) were used to blot for loading controls. Bands were imaged and analyzed with an Alpha Innotech CCD camera and AlphaEaseFC (FluorChem8000) software, respectively.

### IGF-I measurements

Serum was stored at −80°C until assayed for hormones. IGF1 was measured with enzyme-linked immunosorbent assay kits in accordance with the manufacturer's (Diagnostic Systems Laboratories) protocol.

### Histology and immunohistochemistry analysis

With the exception of testes that were fixed in Bouin's solution, all tissues were fixed in 4% paraformaldehyde, processed, embedded in paraffin, and sectioned (5 µm). To detect the expression pattern of SAFB2 and SAFB1 in tissues, the newly generated N-terminal polyclonal SAFB2 (see above) and polyclonal SAFB1 (B1-1753) antibodies were used at a dilution of 1:150 and 1:500, respectively. Sertoli cells were stained with WT1 monoclonal antibody 6F-H2 (DakoCytomation). An anti-AR antibody (Santa Cruz sc-816) was used for IHC at a dilution of 1:50. β-galactosidase immunofluorescent staining was performed as described previously (Ismail et al., 2002). Briefly, testes were embedded in OCT medium (Sakura Finetechnical Co., Ltd, Tokyo, Japan), cryosectioned at 20 µm, and fixed for 15 min with 2% formaldehyde, 0.2% glutaraldehyde, 0.02% Nonidet P-40, then washed and incubated overnight at 37°C in x-gal staining solution [1.3 mM MgCl_2_, 15 mM NaCl, 5 mM K_3_Fe(CN)_6_, 5 mM K_4_Fe(CN_6_).3H_2_O, 0.02% Nonidet P-40, 44 mM HEPES (pH 7.9), and 0.05% (wt/vol) 4-chloro-5-bromo-3-indolyl-β-D-galactopyranoside]. The sections were washed and counterstained with Nuclear Fast Red. All images were captured using an Olympus IX70 inverted scope under a 40× objective with a CCD camera.

### AR reporter assay

LNCaP and COS7 cells were maintained in RPMI 10% FBS, and 293T cells were maintained in DMEM 5% FBS, and all cells were plated for experiments in phenol red free RPMI+10% CSS. Lipofectamine LTX/Plus (Invitrogen) was used to transiently transfect 1 μg of hSAFB2 or empty vector (pcDNA3.1), 100 ng of androgen response element (ARE), and/or 10 ng AR into LNCaP cells for 24 h. All cells were transfected with 10 ng of null-renilla. Cells were then treated with R1881 (1 nM) or vehicle for 24 h. A dual luciferase reporter assay (Promega) was then conducted as per the manufacturer's instructions.

### Statistical analysis

Number of pups, number of embryos, serum IGF1 levels, AR-positive cells and body weight were assessed using descriptive statistics, including *t*-tests, Wilcoxon rank-sum tests, ANOVA and Chi-square tests. Number of samples and statistical analyses are indicated in figures and/or figure legends.

## Supplementary Material

Supplementary Material
