# Supplementary material for: Scaffold attachment factor B2 (SAFB2)-null mice reveal non-redundant functions of SAFB2 compared with its paralog, SAFB1
Source: Dis Model Mech. 2015 Sep 1;8(9):1121–7. doi: 10.1242/dmm.019885 (PMC4582101; doi:10.1242/dmm.019885)
Supplement: Supplementary Material [file supp_8_9_1121__index.html]

Supplementary Material 

# Scaffold attachment factor B2 (*SAFB2*)-null mice reveal non-redundant functions of *SAFB2* compared with its paralog, *SAFB1*

## DMM019885 Supplementary Material

- Supplementary Material
