# Supplementary material for: Scaffold attachment factor B2 (SAFB2)-null mice reveal non-redundant functions of SAFB2 compared with its paralog, SAFB1
Source: Dis Model Mech. 2015 Sep 1;8(9):1121–7. doi: 10.1242/dmm.019885 (PMC4582101; doi:10.1242/dmm.019885)
Supplement: Supplementary Material [file supp_019885_DMM019885supp.pdf]

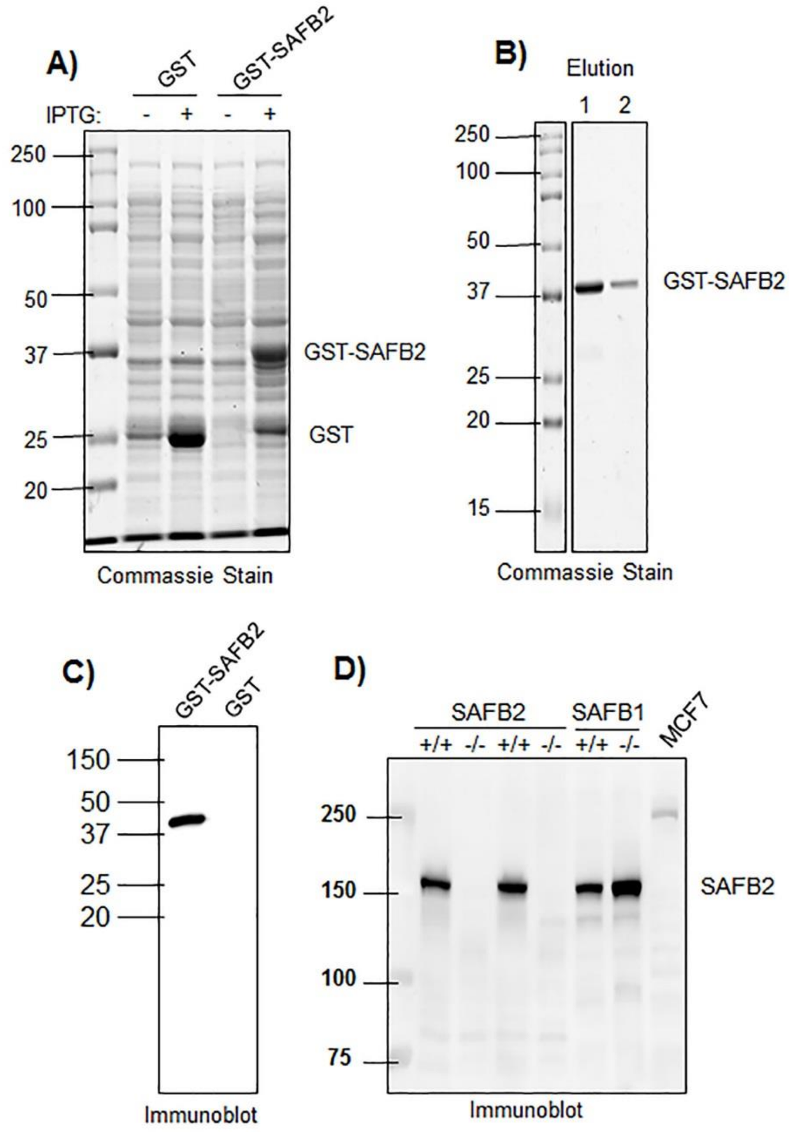

Supplementary Figure S1: **Developing and validating SAFB2 antibodies.** **A)** IPTG-induced expression of recombinant GST only, and GST-SAFB2 fusion protein (~39 Kd) resolved by SDS-PAGE on a 10% gel stained with commassie blue. **B)** Purified recombinant GST-SAFB2 fragments after protein isolation resolved by SDS-PAGE on a 12% gel stained with commassie blue. **C)** Immunoblotting of GST-SAFB2 fusion protein using SAFB2 N-terminal antibody (see Materials and Methods for details on antibody generation). **D)** Immunoblot of protein lysate from SAFB2 $^{+/+,\ +/-,\ -/-}$ and SAFB1$^{+/+,\ +/-,\ -/-}$ animal tissues using SAFB2 antibody.

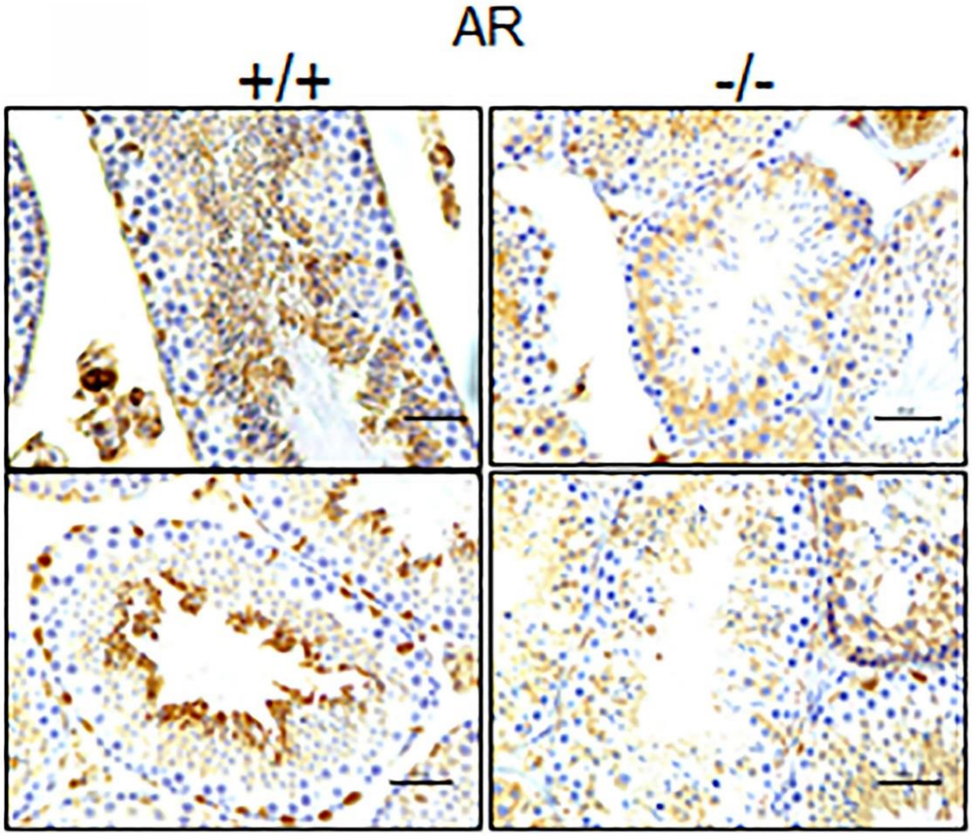

Supplementary Figure S2: **AR expression in SAFB2$^{+/+}$ and SAFB2$^{-/-}$.** Representative images of AR IHC counterstained with hematoxylin in mature (1 year) SAFB2$^{+/+}$ and SAFB2$^{-/-}$ mouse testes (scale bar = 40 um).

SUPPLEMENTARY TABLE S1

Primer sequences for generation of SAFB2 targeting vector

Primer Set 1: 5'arm	5'-CCCTCGAGCATTTAAGGAGGAAGGACAAGAAC-3'
	5'-CCCTCGAGGCCACGTACTCTTTACTGTCATCA-3'
Primer Set 2: 3' arm	5'-CTCGCGGCCGCAGTCGTCAGACAGGAGAGCATGTGAC-3'
	5'-CTCTCCCGCGGCTCCTTGATCTTGTCGAATGACAG-3'

Primer sequences for validation of SAFB2 knockout

Exon coverage	Target Gene	Primer Set
18-21	SAFB2	5'-ATGGCTACAGCTCTGACAAGAAGTT-3'
		5'-AGTCTACCACTGGGCACAATATAGC-3
4-7	SAFB2	5'-GATTCTTCAGAGTTCACAAGACAGG-3'
		5'-TGTCGAGTATTTTCTCATTTTCTGG-3'
9-11	SAFB1	5'-GAGAAAGGTGAGAGAAAAGACGATG-3'
		5'-AGTCTCTGGACTTTCGAGACTCTTT-3'
1-4	SAFB1	5'-ATGAAATTGAAGTCACCTCAGAATG-3'
		5'-TGTTAACGGTGTCCTTGTCATCTAT-3'
	β-actin	5'-TCACCCACACTGTGCCC-3'
		5'- CCAAGATGGAGCACCG-3

Sequence of Southern Blot probe

TGACGGTAGTGACCCTCACATGGCAGAGAACACCAAGGCCTTGGCTGTACAT
GGACCTTTGGCCTCAGCAGACCCTGGTGGGAGGGCAGGGACTCAGCCAAGGC
CTCTGGGGTCTGGACAGGAGGCCAGAGGCTGAGTATGGGCAGCACTCAGGCC
ACAAACTGAGGTTTCAGCCACCCGTGTGCAGGGAGCGGGAGCAGCGAGAGC
GGGAGCAGCGTCTGGATGCCTTCCAGGAGCGACGGGAGAAGGCTCGCTTGCA
GCGGGAACGGATGCAGCTCCAGTGCCAGCGGCAGCGGTTGGAGCGGGAGCG
GCTGGAGCGGGAGAGGCTGGAGCGGGAGCGCATGAGAGTGGAGCGTGAAAG
GCGCAAGGAACAGCAGCGCATTATGCGTGAGCGGGAGGAGCTGCGGCGGCA
GCAAGAACAGCTGCGTGCTGAGCAGGAACGGCGGGCACTGCGCAGACCCTA
CGACCTGGATGCTAGGTGAGTACAGATACCAGTCCTGCCTCCTCTCCCCAGA
GGACAAGCATGTAGTGCTTTTCATTGGCCAGCACCTTGTATTGCCCAGCCACT
CGGGGAGAGGCTTGAGGATCCACTGTTGTG

Sequence of Northern Blot probe

aagaacagct gcgtgctgag caggaacggc gggcactgcg cagaccctac gacctggatg
ctaggagaga cgatggctac tggccagaag ggaagcgtgc agccttagag gacagatacc

gagactttcc acggccagat caccgcttcc atgactttga tcaccgagat cgtggccatt
accaggagca tgtcatagac aggcgggatg ggtccagaac cagagtggag gagcgggatg
ggcagtacta cccagatgac cagcacagcc atggaaggct cctagagcac catgcttggg
attccggaga cggctggcat ggctacagct ctgacaagaa gttgaatgaa ggccaagggc
taccccctcc ccccagggtc agccgagagt gggcggagca cagctcacag ttggaggagc
agcaggttcc tgtctggcac agtgctgtgg acacaaacat gacgggccat gaacacatac
ggtggcgagg tgctgagcgg ggccttgcag gaggacctgg gcatgggcat gtggcagcag
gccggggtgg catggctggg caaggcagct ttgcacacgg tggacattcc cagggctata
ttgtgcccag tggtagact

Primer sequence to amplify cDNA encoding SAFB2 aa 105 to 199 for generation of SAFB2 N-terminal antibodies. The mB2 peptide sequence encoded is:
RYGQDGVVILQSSQDRDTMDTGVPDGMEAEDLSVPCLGKADTVNQILHAFDDS
KEYVAAQLGQLPAQLLKHAVDEEVFKNTLEASVSDLKVTLAD

5' primer	GTCGGATCCAGATATGGGCAGGATGGGGTTGTGA
3' primer	GTCGAATTCTCAATCAGCCAGAGTTACTTTAAGGTCTG
